# Study and Reuse of Silicone Implants After Radiotherapy

**DOI:** 10.3390/ma19091798

**Published:** 2026-04-28

**Authors:** Aleksandra Kozłowska, Marta Worzakowska, Bogdan Tarasiuk, Maria Piziorska, Beata Podkościelna

**Affiliations:** 1 Department of Radiotherapy, University Clinical Hospital No. 1, 13 Radziwiłłowska Street, 20-080 Lublin, Poland; olla.kozlowska@gmail.com (A.K.); maria.piziorska@gmail.com (M.P.); 2 Department of Polymer Chemistry, Faculty of Chemistry, Institute of Chemical Sciences, Maria Curie-Sklodowska University, Gliniana St. 33, 20-614 Lublin, Poland; marta.worzakowska@mail.umcs.pl (M.W.); bogtar2020@wp.pl (B.T.)

**Keywords:** breast silicone implants, post-mastectomy radiotherapy, reuse of breast implants, TG/DTG/FTIR

## Abstract

Studies on the influence of radiotherapy on the structure, thermal properties, and thermo-oxidative decomposition of breast silicone implants were conducted. Additionally, the potential use of breast silicone implant waste as a component in layered composites was investigated. ATR-FTIR, DSC, and TG/DTG/FTIR analyses confirmed that radiation does not affect the structure, thermal properties, or oxidative decomposition behavior of the shell and gel layers of breast silicone implants. The conducted tests demonstrated the successful fabrication of composite materials using a PUM matrix and breast silicone implant waste. The presence of the PUM matrix in the manufactured composites influenced the crystallization and melting behavior of the silicone phase. Moreover, the incorporation of a silicone implant waste layer into the composites increased their thermal stability while decreasing the glass transition temperature, storage modulus and hardness compared to neat PUM. The type of implant waste layer used (shell or gel) in the preparation of the PUM composites did not significantly affect the melting and glass transition temperatures, thermal stability, or oxidative decomposition behavior of the newly developed materials. As demonstrated, new layered composite materials based on silicone implant waste (shell and gel), with properties valuable for practical applications, were successfully developed.

## 1. Introduction

Immediate implant-based reconstruction after mastectomy is now a common clinical practice. Indications for post-mastectomy radiation therapy (PMRT) remain unchanged by the presence of implants and include high-risk factors such as tumor size > 5 cm, chest wall or skin involvement, positive margins, and ≥4 positive lymph nodes. Decisions in cases with 1–3 positive lymph nodes are increasingly individualized. Managing PMRT alongside immediate reconstruction requires balancing oncological safety with esthetic outcomes [1,2,3]. While conventional fractionation schedules (50 Gy in 25 fractions) are still used, moderate hypofractionation (40–42.5 Gy in 15–16 fractions) has become the standard of care, demonstrating comparable efficacy without increasing implant-related complications [3,4]. In prepectoral reconstruction, the implant is placed above the pectoral muscle, offering more natural breast contours, reduced postoperative pain, and faster recovery compared to the submuscular approach. However, careful patient selection is essential due to thinner soft-tissue coverage and an increased risk of post-radiation rippling and ischemia. An ideal candidate for the prepectoral technique is a non-smoker with a moderate body mass index (BMI). Acellular dermal matrices (ADMs), used as biological scaffolds, help reduce animation deformity (involuntary breast movement during muscle contraction) associated with radiation-induced fibrosis, improve esthetic outcomes, and enhance tissue integration [5,6]. In clinical practice, complications associated with PMRT in patients undergoing prepectoral reconstruction are classified as acute or late effects. Early complications occur during treatment or within six months after radiotherapy and include dermatitis, implant exposure, seroma, and fat necrosis. Late complications, occurring after six months, include capsular contracture, telangiectasia, and skin atrophy or fibrosis [7]. To minimize treatment-related morbidity, advanced radiation delivery techniques are essential. Volumetric modulated arc therapy (VMAT) enables highly conformal dose distribution, shaping isodose curves to minimize irradiation of the heart and lungs while maintaining dose homogeneity within the clinical target volume. To address the risk of skin recurrence, which is often thin and stretched, bolus materials (tissue-equivalent layers) are used to increase the surface dose. Furthermore, deep inspiration breath hold (DIBH) is routinely applied in left-sided cases to increase the distance between the heart and the radiation field, thereby reducing long-term cardiotoxicity. Patients should be informed about the importance of gentle daily skin care, prophylactic moisturizing, loose clothing, and sun protection during and after radiotherapy [2,3].

Modern silicone breast implants are extensively tested and considered safe; however, they are not lifetime devices. Radiotherapy after mastectomy clearly increases the risk of implant-related complications. Implants are also removed or replaced for esthetic and personal reasons, including changes in patient preference or dissatisfaction with appearance. In addition, replacement is often recommended based on duration of use, as implants typically have a lifespan of approximately 10–15 years. Prolonged contact with biological tissues may lead to material aging and changes in properties, necessitating replacement or removal [6,7]. Implants removed from one patient are not reused in another, due to sterility concerns, risk of complications, and safety, hygiene, and ethical considerations. They are certified single-use medical devices. Silicone breast implants removed for medical or esthetic reasons are treated as medical waste and disposed of, most commonly via thermal treatment. The most widely used method is high-temperature incineration in specialized facilities, which leads to the complete decomposition of the silicone material.

Surgical procedures involving breast implants generate significant amounts of medical waste, particularly in the form of single-use plastics. Approximately 4 kg of plastic waste is produced during an implant replacement [8]. Meanwhile, attention is drawn to the significant carbon footprint of breast reconstruction procedures involving implants, mainly due to the production and transportation of medical equipment, and the use of single-use materials [9]. Therefore, there is a growing emphasis on the need to implement strategies that reduce waste and improve the sustainable management of resources in plastic surgery [10]. Despite growing interest in sustainability issues, there are relatively few reports in the scientific literature regarding the recyclability of used medical implants. While these materials cannot be reused for medical applications due to biological safety concerns, they could potentially be used as a source of raw materials for producing new materials for non-medical applications [11]. In this context, this article presents a new approach involving using used implants as a component in producing new composite materials.

In the present work, we investigate the effects of ionizing radiation on silicone breast implants and propose the reuse of silicone implant waste obtained after radiotherapy or explantation for personal reasons (in cases not previously exposed to radiotherapy) as a component of layered composites. In our study, both layers of silicone implant waste, the outer shell and inner gel, were used to fabricate novel composite materials. The structure of the implants and the prepared composites was confirmed using attenuated total reflection Fourier transform infrared spectroscopy (ATR-FTIR). The effects of ionizing radiation on glass transition temperature, melting and crystallization behavior, thermal stability in inert and oxidative atmospheres, and thermal degradation pathways under heating in synthetic air were evaluated. Furthermore, the influence of the silicone layer type (shell or gel) on the thermal properties, thermal stability, viscoelastic properties, hardness and thermo-oxidative degradation behavior of the prepared composites was investigated using differential scanning calorimetry (DSC) and thermogravimetric/derivative thermogravimetric analysis coupled with Fourier transform infrared spectroscopy (TG/DTG/FTIR), dynamic mechanical analysis (DMA) and Brinell hardness.

## 2. Materials and Methods

### 2.1. Materials

Commercially available gel-filled breast implants, consisting of silicone gel (polysiloxane) enclosed in a silicone elastomer shell (Mentor, OH, USA), were used. 2,4-diisocyanato-1-methylbenzene, poly(oxypropylene)diol (Mw = 2000 g/mol), 2-hydroxyethyl methacrylate, 3-(trimethoxysilyl)propyl methacrylate, 2-ethylhexyl acrylate, 2,2-dimethoxy-2-phenylacetophenone (photoinitiator), and dibutyltin dilaurate were purchased from Sigma-Aldrich (Darmstadt, Germany). The liquid composition was cured using TL-K 40W/10-R UV-A lamps (Philips, Amsterdam, the Netherlands), emitting radiation in the wavelength range of 315–380 nm, with a UV-A irradiance of 8.0 W (0 h, IEC-International Electrotechnical Commission).

### 2.2. Preparation of the Composites

#### 2.2.1. Synthesis of Oligomeric Urethane-Methacrylate Resin (OUM)

**Figure 1:**
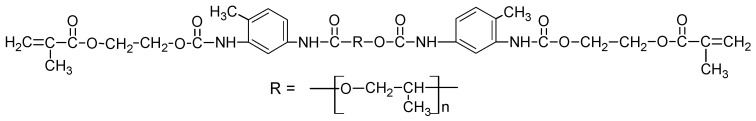
Chemical structure of resin oligo(urethane–methacrylate) (OUM).

In the first step, an oligomeric urethane-methacrylate resin (OUM) was synthesized by reacting 0.05 mol of 2,4-diisocyanato-1-methylbenzene with 0.025 mol of poly(propylene)diol. The reaction was carried out at 80 °C for 5 h. The resulting urethane prepolymer was then cooled to 50 °C, followed by the addition of 0.05 mol of 2-hydroxyethyl methacrylate and 22.0 g of 2-ethylhexyl acrylate, used as a reactive diluent. The reaction was continued at 55 °C for 2 h. Subsequently, the mixture was again cooled to 50 °C, and 0.20 g of dibutyltin dilaurate was added as a catalyst. The system was stirred for an additional 2 h to promote and complete the formation of the urethane–methacrylate oligomer. Detailed physicochemical data are provided in Ref. [12]. Figure 1 presents the chemical structure of the obtained oligo(urethane-methacrylate) resin.

#### 2.2.2. Synthesis of New Compositions Based on Waste Breast Implants

**Figure 2:**
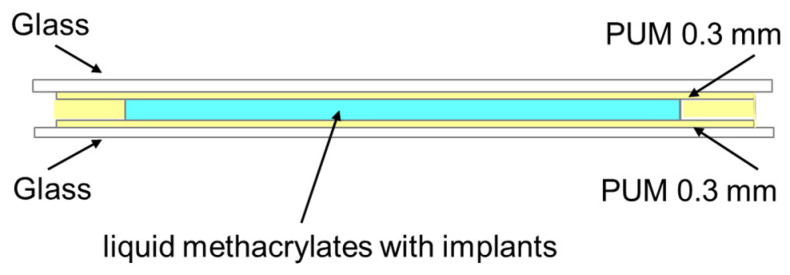
Schematic diagram of the mold for preparing the silicone recycling composite.

**Figure 3:**
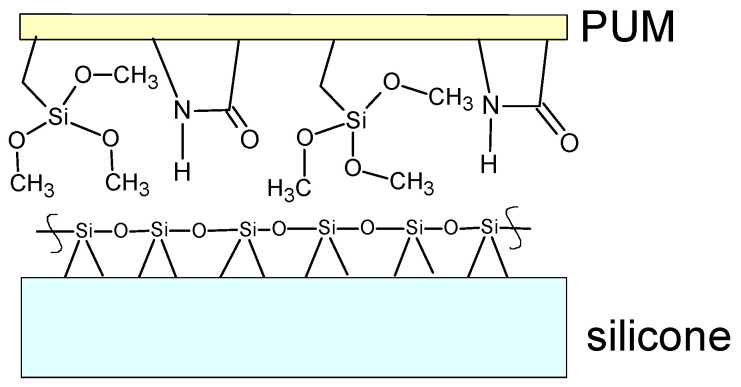
Chemical structure of functional groups at the PUM polymer–silicone interface.

In the second step, a urethane–methacrylate composition (PUM) was prepared by mixing the following components: oligo(urethane–methacrylate) resin (OUM), 3-(trimethoxysilyl)propyl methacrylate (10 wt%), and the photopolymerization initiator 2,2-dimethoxy-2-phenylacetophenone (4 wt%). Two types of composites were prepared depending on the type of silicone from the breast implants used, namely the shell or the gel fraction. The procedure was as follows: two thin PUM films were initially cured under UV irradiation for 2 min. Subsequently, a layer consisting of PUM, gel, or silicone shell containing liquid methacrylates was placed between two glass plates and exposed to UV irradiation for 10 min. After curing, the solid samples were subjected to further testing. A schematic diagram of the mold polymerization process is shown in Figure 2. The chemical structure of the functional groups at the PUM polymer–silicone interface is presented in Figure 3.

#### 2.2.3. Methodology of Irradiation of Silicone Breast Implants

The implant was irradiated using a standard protocol commonly applied in clinical practice. The treatment plan was developed using the MONACO treatment planning system. A total dose of 50 Gy was delivered in 25 fractions of 2 Gy using 6 MV photon beams generated by a Versa HD linear accelerator (Elekta, Stockholm, Sweden). The volumetric modulated arc therapy (VMAT) technique was employed.

### 2.3. Methods

#### 2.3.1. Attenuated Total Reflection Fourier Transform Infrared Spectroscopy (ATR-FTIR) Measurements

ATR-FTIR spectra of the investigated silicone breast implants (before and after radiotherapy) and the prepared layered composites were recorded using a Tensor 27 FTIR spectrometer equipped with a diamond ATR crystal (Bruker, Bremen, Germany). Each spectrum was collected over the range of 600–4000 cm^−1^ with a resolution of 4 cm^−1^ and 64 scans.

#### 2.3.2. Differential Scanning Calorimetry (DSC) Measurements

DSC measurements were performed using a DSC 204 Phoenix calorimeter (Netzsch, Selb, Germany). Samples with a mass of approximately 10 mg were placed in aluminum crucibles with pierced lids and analyzed under an inert atmosphere (argon, flow rate: 30 mL/min). The heating and cooling rate was 10 °C min^−1^. The DSC procedure was as follows: the sample was first cooled to −150 °C, then heated to 150 °C, cooled again to −150 °C, and finally reheated to 150 °C. Each measurement was repeated three times, and the values represent the arithmetic mean of these measurements. Characteristic temperatures, including the glass transition temperature (*T*_g_), crystallization temperature (*T*_c_), and melting temperature (*T*_m_), as well as the corresponding enthalpies of crystallization (Δ*H*_c_) and melting (Δ*H*_m_), were determined based on the DSC curves.

#### 2.3.3. Thermogravimetric/Derivative Thermogravimetric (TG/DTG) Measurements

Thermogravimetric (TG/DTG) analysis was performed to evaluate the thermal properties of the tested silicone samples and the prepared composites using an STA 449 Jupiter F1 instrument (Netzsch, Selb, Germany). In a typical experiment, approximately 10 mg of sample was placed in an open corundum crucible and heated from 40 °C to 1000 °C at a heating rate of 10 °C min^−1^. All analyses were carried out under two different furnace atmospheres: inert (helium, flow rate: 40 mL/min) and oxidative (synthetic air, flow rate: 100 mL/min). Each measurement was repeated three times, and the results are the arithmetic mean of these measurements. Based on the TG/DTG curves, the following parameters were determined: the initial decomposition temperature corresponding to a 5% mass loss (*T*_5%_), the temperatures of the maximum decomposition rate (*T*_max_), mass losses at individual decomposition stages (Δ*m*), and the residual mass at 1000 °C (*m*_r_).

#### 2.3.4. Simultaneous Thermogravimetric/Derivative Thermogravimetric Analysis Coupled with Fourier Transform Infrared Spectroscopy (TG/DTG/FTIR) Measurements

Simultaneous TG/DTG/FTIR analysis was carried out using an STA 449 Jupiter F1 instrument (Netzsch, Selb, Germany) coupled online with a TGA 585 FTIR spectrometer (Bruker, Selb, Germany). FTIR spectra of gaseous products evolved during the oxidative decomposition of the tested silicone implants and the prepared composites were collected to identify the emitted compounds and assess their potential toxicity during disposal via combustion. The spectra were recorded in the wavenumber range of 600–4000 cm^−1^ with a resolution of 4 cm^−1^ and 32 scans per spectrum.

#### 2.3.5. Dynamic Mechanical Analysis (DMA) Measurements

DMA was performed on a DMA Q 800 (TA Instruments, New Castle, DE, USA). Tests were con-ducted using a tension clamp. Measurements for all samples were carried out from −150 °C up to the temperature at which the samples became too soft to be tested. A constant heating rate of 3 °C min^−1^ and an oscillation frequency of 1 Hz were applied. Rectangular samples with dimensions of 10 mm in length, 6 mm in width, and 1 mm in thickness were used. The storage modulus (*E*_20°C_) and maximum tangent delta (tg *δ*) were evaluated.

#### 2.3.6. Brinell Hardness Measurements

The Brinell hardness (HK) was determined using an HPK hardness tester (Schifferstadt, Germany). In this procedure, a steel ball indenter with a diameter of 5 mm was hydraulically pressed into the material surface under a load (*F*) of 13.5 kgf, applied for 60 s. The resulting indentation depth (*h*), expressed in centimeters, was measured. Ten measurements were performed for each sample. The HK values are the arithmetic mean of these measurements with the calculated standard deviation. The Brinell hardness (HK), expressed in megapascals (MPa), was then calculated using the following equation:

$$HK\;[MPa]=\frac{F}{1.57\cdot h\cdot 0.098066}=F_{1}\cdot 0.098066$$

where *F* is the applied load (13.5 kgf) and *h* is the indentation depth (cm), with *F*_1_ = *F*/(1.57 × *h*).

#### 2.3.7. Surface Morphology Characterization

Morphological images and surface roughness were determined for each sample using a Contour GT-K1 optical profilometer (Veeco Instruments, Inc., Plainview, NT, USA) which has a very high level of accuracy in the size range from subnanometers to 10 mm. The experiments were repeated three times for each sample. The average roughness (Ra) parameter was determined.

## 3. Results

The investigated silicone breast implants, both after radiotherapy and those not previously subjected to radiotherapy (referred to as “before radiotherapy”), consist of two distinct layers. The outer layer (shell) is organoleptically harder, yet elastic, and less transparent. In contrast, the inner layer (gel) is softer, more fluid, and more transparent. Due to the layered structure of the implants, both the shell and gel were analyzed before and after radiotherapy. The shell before radiotherapy is denoted as (a), and the gel before radiotherapy as (b). The corresponding layers after radiotherapy are denoted as (a′) and (b′). Furthermore, both implant layers were used as components in composites with PUM, denoted as (c).

### 3.1. ATR-FTIR Results

#### 3.1.1. ATR-FTIR Spectra of Silicone Breast Implants

**Figure 4:**
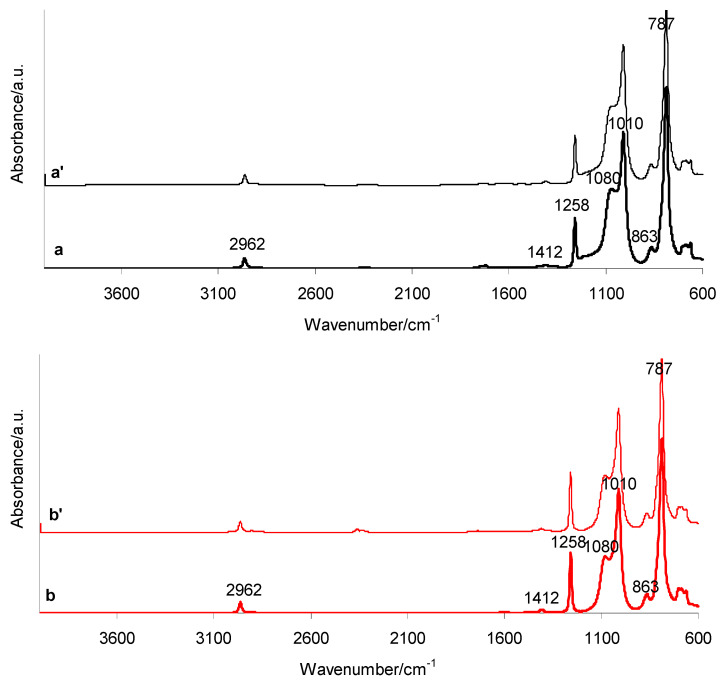
ATR-FTIR spectra of silicone breast implants: shell before (a) and after radiotherapy (a′), and gel before (b) and after radiotherapy (b′).

Figure 4 presents the ATR-FTIR spectra of the investigated silicone breast implants before (a, b) and after (a′, b′) radiotherapy. The spectra include both the shell (a, a′) and gel (b, b′) layers of the implants. All FTIR spectra exhibit characteristic absorption bands corresponding to asymmetric stretching vibrations of –CH_3_ in Si–CH_3_ groups (2905 and 2962 cm^−1^), asymmetric deformation vibrations of –CH_3_ (1412 cm^−1^), symmetric deformation vibrations of –CH_3_ in Si–CH_3_ (1258 cm^−1^), and asymmetric stretching vibrations of Si–O–Si in the silicone backbone (1010 and 1080 cm^−1^). Additionally, rocking vibrations of Si–CH_3_ (863 cm^−1^) and coupled vibrations involving Si–C stretching in Si–CH_3_ and –CH_3_ rocking (787 cm^−1^) are observed. Importantly, both the shell and gel spectra, recorded before and after radiotherapy, show absorption bands in the same wavenumber regions. This indicates that radiotherapy exposure does not induce detectable structural changes in the investigated implants. Furthermore, the ATR-FTIR analysis confirms that the main component of the tested silicone breast implants, in both shell and gel layers, is polydimethylsiloxane (PDMS) [13,14,15,16,17,18].

#### 3.1.2. ATR-FTIR Spectra of the Prepared Composites

**Figure 5:**
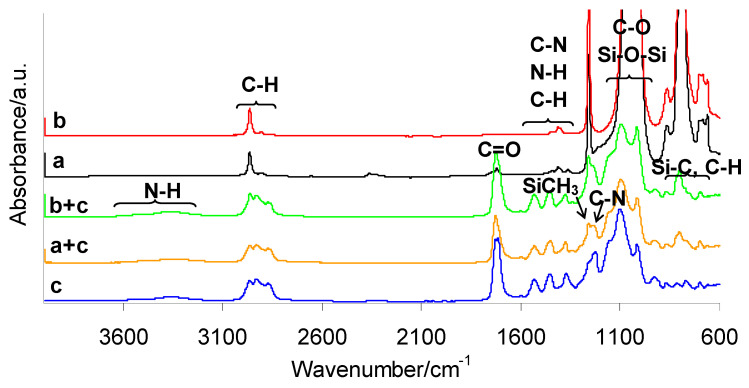
ATR-FTIR spectra of silicone breast implants: shell (a), gel (b), PUM (c), and prepared composites: shell–PUM (a + c) and gel–PUM (b + c).

Due to the lack of structural changes in silicone breast implants after radiotherapy, it can be assumed that these materials are not subjected to degradation under ionizing radiation. Therefore, both pre- and post-radiotherapy silicone breast implants can be used for composite preparation without significant changes in their properties. Figure 5 presents the ATR-FTIR spectra of the prepared composites containing the shell fraction of breast implant waste (a + c) and those containing the gel fraction (b + c). For comparison, the spectra of both implant layers (a and b) and the PUM matrix (c) are also shown.

As it is clearly observed, the ATR-FTIR spectra of both PUM and the prepared composites exhibit a broad absorption band in the range of 3500–3100 cm^−1^, which is attributed to N–H stretching vibrations. In addition, a weaker band at 3269–3280 cm^−1^ corresponds to hydrogen-bonded N–H stretching vibrations in both the composites and PUM. The C–H stretching vibrations associated with PUM, silicone implants (C–H in Si–CH_3_), and the composites (C–H in both Si–CH_3_ and PUM) appear in the range of 2968–2864 cm^−1^. The absorption band at 1722 cm^−1^ indicates the presence of C=O stretching vibrations in both the composites and the neat PUM. Bands observed at 1523–1570 cm^−1^ are attributed to C–N stretching vibrations and N–H bending vibrations in the structure of PUM and the composites. Bending vibrations of C–H groups appear in the region of 1360–1440 cm^−1^. The FTIR spectra of the composites also show two characteristic maxima at 1228 cm^−1^ and 1255 cm^−1^, corresponding to C–N stretching in urethane groups and deformation vibrations of –CH_3_ in Si–CH_3_, respectively. Intense absorption bands in the range of 1010–1140 cm^−1^ are associated with C–O stretching vibrations of ether groups and Si–O–Si stretching vibrations of the silicone backbone. Additionally, rocking vibrations of Si–CH_3_ (863 cm^−1^) and coupled vibrations involving Si–C stretching in Si–CH_3_ and –CH_3_ rocking (787 cm^−1^) are clearly observed [17,18,19]. The presence of all these characteristic bands confirms the expected chemical structure of the prepared materials and the successful fabrication of composites based on PUM and silicone breast implant waste.

### 3.2. DSC Results

**Figure 6:**
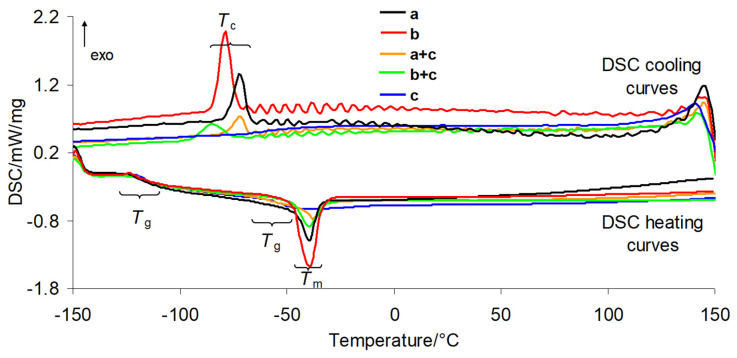
DSC curves for silicone implants: shell (a), gel (b), PUM (c) and the prepared composites: shell–PUM (a + c) and gel–PUM (b + c).

DSC analysis of the investigated silicone breast implants before and after radiotherapy, as well as of the prepared composites, enabled the determination of the glass transition temperature (*T*_g_), melting temperature (*T*_m_), crystallization temperature (*T*_c_), enthalpy of fusion (Δ*H*_f_), and degree of crystallinity (*χ*). The obtained DSC curves for the shell and gel fractions of the silicone implant waste, PUM, and the prepared composites are shown in Figure 6. The numerical data derived from the DSC curves, including results for implants before and after radiotherapy, are presented in Table 1. The glass transition temperature (*T*_g_) characterizes the transition of the amorphous phase from a rubbery to a glassy state. At *T*_g_, long-range translational and rotational motions of polymer chains become frozen. *T*_g_ is directly related to polymer chain mobility; higher mobility corresponds to lower *T*_g_ values [20]. Based on the DSC results, the polymer chain mobility in the investigated implants can be considered high. The *T*_g_ values for the shell and gel before radiotherapy are comparable, at −123.9 °C (shell) and −121.3 °C (gel), respectively. These values are consistent with literature data [21,22,23,24]. After radiotherapy, no significant changes in *T*_g_ are observed for either layer; any slight variations fall within the experimental error. Therefore, it can be concluded that radiotherapy does not affect the *T*_g_ of the shell and gel layers of silicone implants and does not induce detectable changes in their chemical structure. For the prepared composites, two glass transition regions are observed, corresponding to two *T*_g_ values. The first *T*_g_ appears at −119.4 °C for the shell–PUM composite and −117.6 °C for the gel–PUM composite. The second *T*_g_ occurs at approximately −61.0 °C for both systems. In comparison, the neat PUM matrix exhibits a *T*_g_ of −50.8 °C. The decrease in the second *T*_g_ by approximately 10 °C in the composites compared to pure PUM is most likely due to the plasticizing effect of the siloxane chains present in PDMS silicone implant waste on the polymer matrix, resulting from their high flexibility (*T*_g_ values below at −121 °C). Upon heating, the investigated implants undergo melting at low temperatures, approximately −36.6 to −39.5 °C (shell) and −39.5 to −42.3 °C (gel), regardless of radiotherapy exposure. The corresponding enthalpy of fusion (Δ*H*_m_), determined from the area of the endothermic peaks, is presented in Table 1. Noticeable differences in Δ*H*_m_ between the shell and gel layers are observed. In both irradiated and non-irradiated samples, the shell exhibits lower Δ*H*_m_ values than the gel. Since Δ*H*_m_ reflects the energy required to melt a material, these differences are directly related to structural differences between the two layers [25,26,27,28,29,30]. According to the literature, the shell of silicone implants consists mainly of a highly cross-linked silicone elastomer formed via vulcanization of silicone rubber [31,32]. Its primary component is polydimethylsiloxane (PDMS), as confirmed by ATR-FTIR analysis. In contrast, the gel layer consists of a lightly cross-linked silicone gel (PDMS) with a cohesive consistency and contains relatively mobile polymer chains [31,32]. Silicone implants are designed as amorphous materials at room temperature and at physiological conditions, which ensures their biocompatibility, thermal stability, and flexibility. However, under specific conditions such as low temperature (freezing) and cooling below the melting point, PDMS can undergo processes in which polymer chains organize into regular structures (lamellae), leading to the formation of a crystalline phase. The DSC melting peaks of the silicone implant waste are asymmetric and broadened over a wide temperature range, which is characteristic of macromolecular amorphous systems. Despite their cross-linked structure, reheating after melting induces partial ordering of PDMS chains during cooling at a controlled rate. As a result, a crystallization peak (exothermic recrystallization) is observed below −70 °C. This ordering process depends on the degree of cross-linking, molecular weight, and the presence of fillers. It indicates the solidification of the amorphous phase, suggesting semi-crystalline behavior at low temperatures. Based on the enthalpy of fusion, calculated from the area of the crystallization peak, the degree of crystallinity (*χ*) can be determined. In this context, crystallinity refers to the ability of the material to undergo partial crystallization after melting, despite the cross-linked structure of PDMS-based silicone implants. The enthalpy of fusion is proportional to the degree of crystallinity. According to literature data, the enthalpy of fusion for fully crystalline PDMS (Δ*H*_f_°) is 61.19 J/g [33,34,35,36]. The degree of crystallinity (*χ*) can be calculated using the equation below:

*χ* = (Δ*H*_f_/Δ*H*_f_°) · 100% (1)

The calculated *χ* values indicate lower crystallinity of the implant shell compared to the gel fraction. This directly confirms that the shell and gel exhibit different polymer network structures associated with varying cross-link densities. A higher cross-link density significantly restricts the mobility of PDMS polymer chains and their ability to organize into ordered structures. In general, polymers with lower molecular weight and a lower degree of cross-linking show a greater tendency to crystallize, as shorter chains can move and arrange more easily. This explains the higher crystallization ability of the gel fraction of the silicone implants (Table 1). Based on the results presented in Table 1, it can also be concluded that radiotherapy has no significant influence on the melting and crystallization behavior of the investigated implants, nor on their degree of crystallinity. In contrast, neat PUM does not exhibit melting or crystallization upon heating and cooling; only a glass transition region is observed in the DSC curves. For the composites, however, both melting and recrystallization peaks are observed. The melting of silicone implant phases within the composites occurs at approximately −37.7 °C (shell) and −39.5 °C (gel). These values are comparable to those observed for the neat silicone implant layers. However, within the composites, the silicone phases exhibit significantly lower melting enthalpy (Δ*H*_m_) compared to the pure PDMS layers. Similarly, the enthalpy of fusion (Δ*H*_f_) of the silicone waste is markedly reduced in the composites. This reduction is attributed to the presence of the PUM matrix, which decreases the crystallization ability of PDMS. PUM matrix in the composites acts as a spatial constraint (steric hindrance effect). It limits the mobility of PDMS chains by interfacial interactions, thereby hindering the formation of crystalline siloxane domains and reducing the relative content of PDMS in the overall composite mass [36].

### 3.3. TG/DTG in Inert Atmosphere

**Figure 7:**
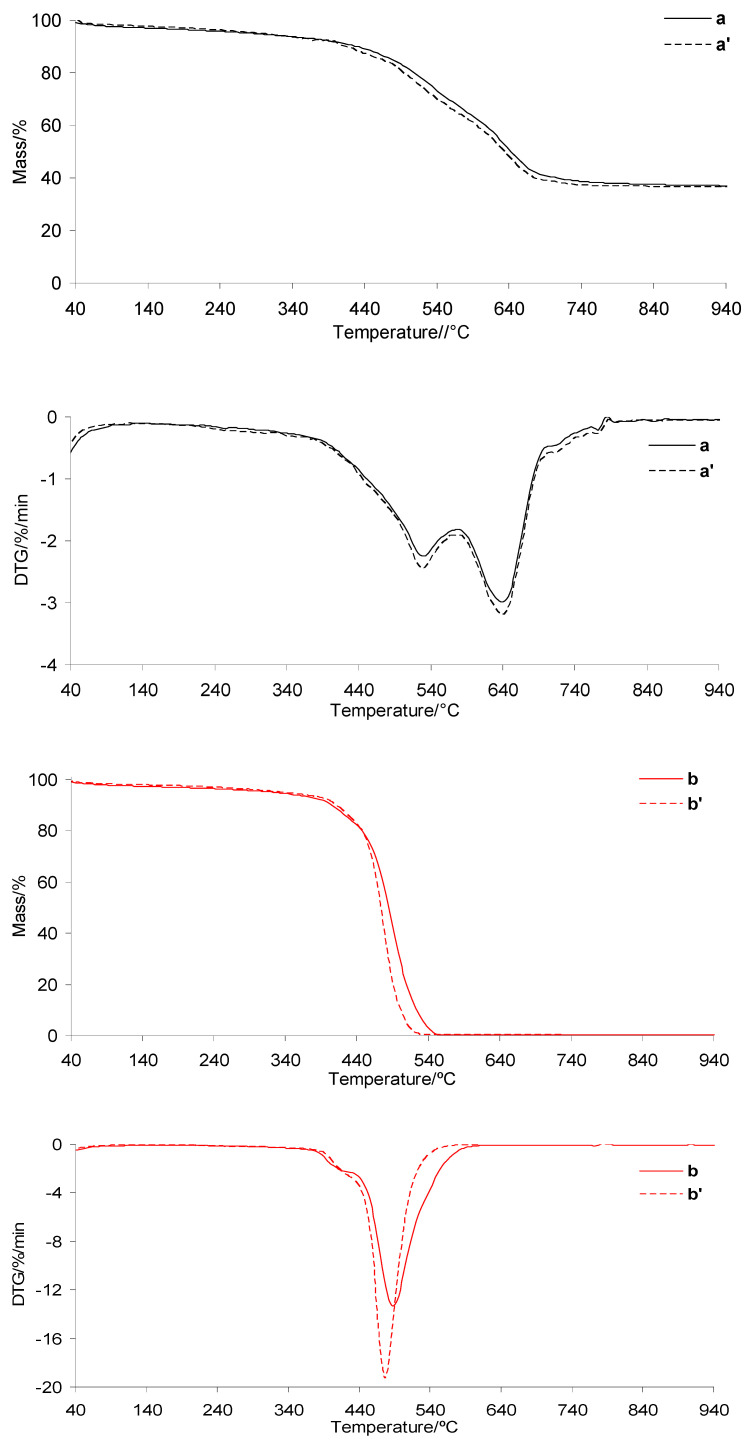
TG/DTG curves of silicone implants: shell before (a) and after radiotherapy (a′), and gel before (b) and after radiotherapy (b′).

**Figure 8:**
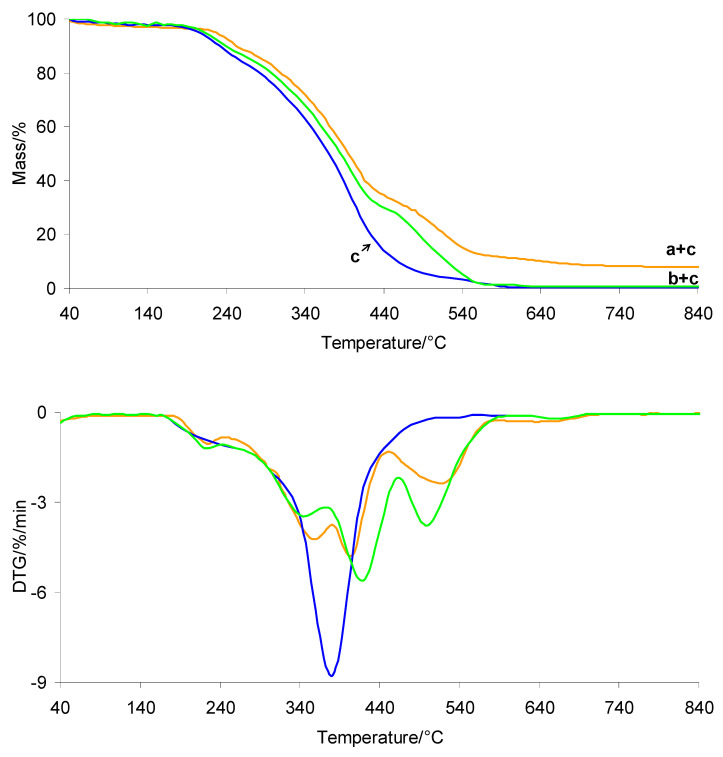
TG/DTG curves of PUM (c) and the prepared composites: shell–PUM (a + c) and gel–PUM (b + c).

The TG/DTG curves of the investigated silicone implants before and after radiotherapy under heating in an inert atmosphere are presented in Figure 7. The TG/DTG curves for PUM and the prepared composites are shown in Figure 8. The corresponding data extracted from the TG/DTG curves are summarized in Table 2. As clearly observed, the thermal decomposition behavior of the silicone implant shell before and after radiotherapy is very similar. The initial decomposition temperature (*T*_5%_) is approximately 330 °C, which is consistent with literature data [37,38,39]. The decomposition occurs in one main stage, consisting of at least three steps, identified as *T*_max1_, *T*_max2_, and *T*_max3_ (Table 2, Figure 7). Upon heating to 1000 °C, the silicone shell does not fully decompose, leaving a residual mass of approximately 36%. This is attributed to its high cross-link density, which promotes the formation of thermally stable intermediates and residues. In contrast, the thermal stability of the gel fraction is not affected by radiotherapy. The *T*_5%_ values are also approximately 330 °C. The decomposition of the gel occurs in one main stage consisting of at least two steps, denoted as *T*_max1_ and *T*_max2_. These decomposition steps occur at lower temperatures compared to those observed for the shell, which is directly related to the lower cross-link density of the gel. As a result, the gel undergoes complete decomposition at approximately 560 °C (Table 2, Figure 7).

The thermal stability of the prepared shell and gel-based composites is approximately 104–108 °C lower than that of the neat silicone implant layers, reaching values of 222.5–226.8 °C (Table 2 and Figure 8). However, it remains approximately 27–31 °C higher than that of the pure PUM matrix. This behavior is directly related to the presence of highly thermally stable silicone implant waste and to interfacial interactions between the shell/gel phases and the PUM matrix.

In addition, the thermal decomposition of the composites proceeds in several poorly resolved stages with *T*_max_ values summarized in Table 2. The first (*T*_max1_) and second (*T*_max2_) stages occur in a temperature range similar to that of neat PUM, indicating that these steps are associated with the degradation of chemical bonds in the PUM matrix. Above 380 °C, further degradation of the composites is primarily governed by pyrolysis of bonds within the silicone shell or gel phases, as well as decomposition of PUM residues. This process leads to the release of volatile products and a significant reduction in residual mass at 1000 °C, particularly for the shell-based composites.

### 3.4. TG/DTG in Oxidative Atmosphere

**Figure 9:**
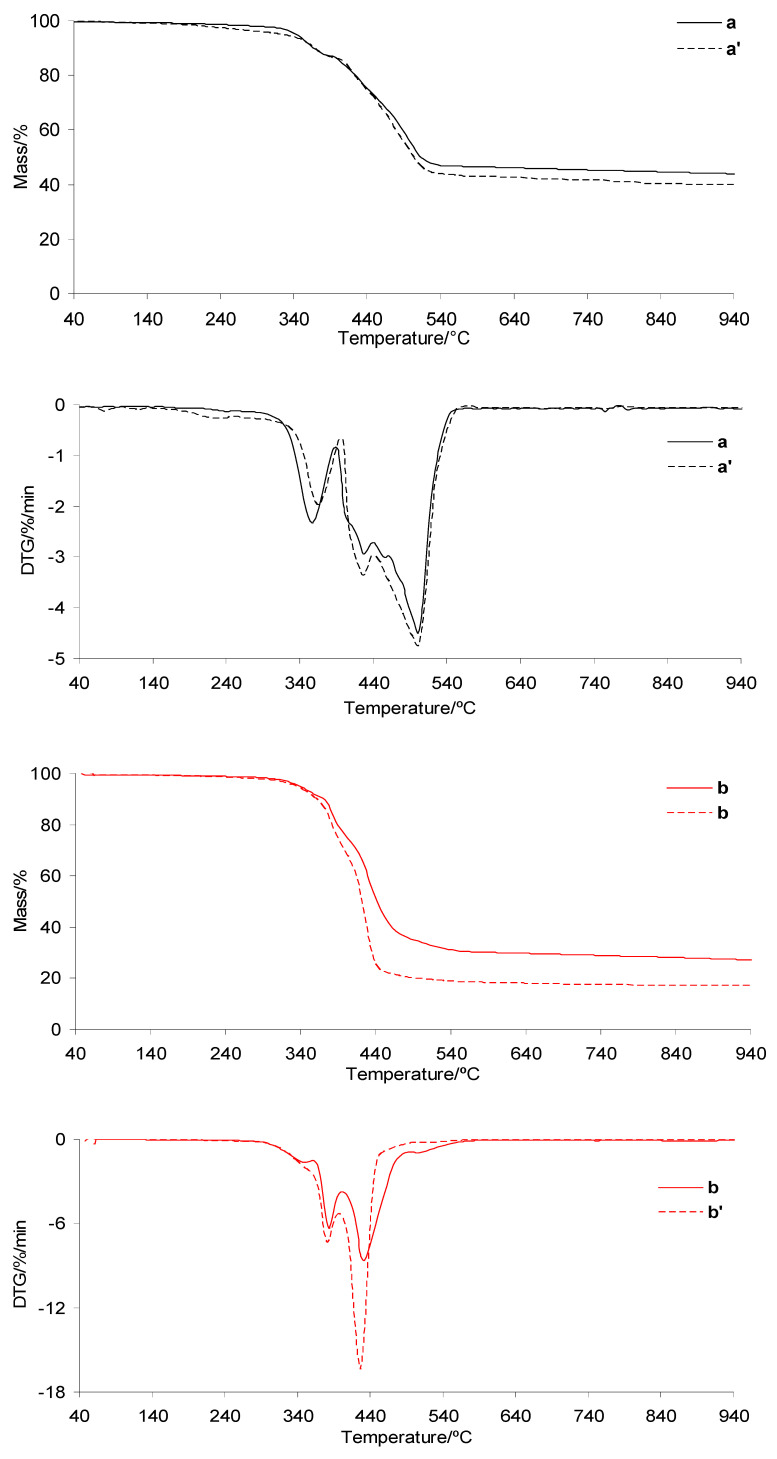
TG/DTG curves of silicone implants: shell before (a) and after radiotherapy (a′), and gel before (b) and after radiotherapy (b′) in oxidative atmosphere.

**Figure 10:**
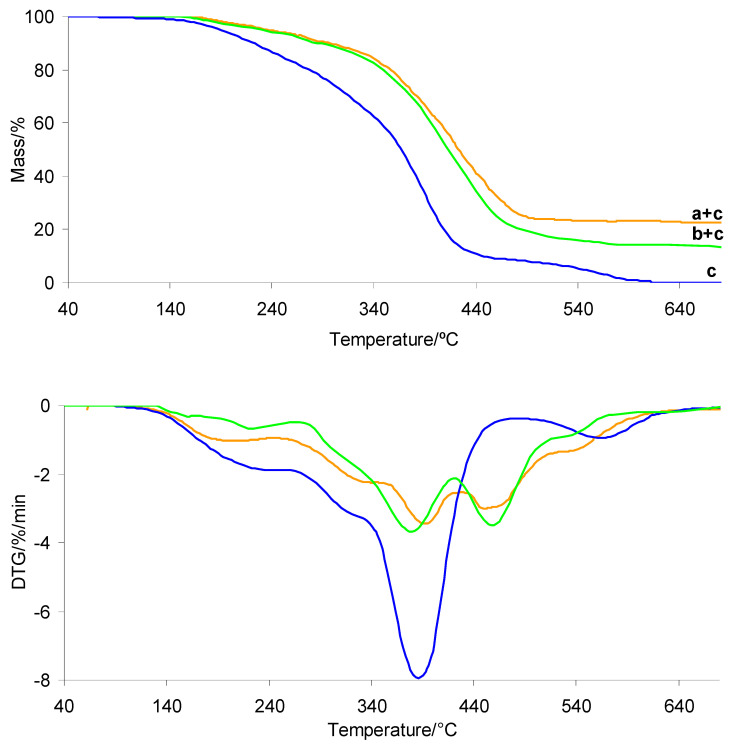
TG/DTG curves of PUM (c) and the prepared composites: shell–PUM (a + c) and gel–PUM (b + c).

The TG/DTG curves recorded during heating of the investigated silicone implants (shell and gel) in an oxidative atmosphere are presented in Figure 9. The TG/DTG curves of PUM and the prepared composites are shown in Figure 10. The corresponding data are summarized in Table 3. In the presence of air, the thermal stability of both silicone implant layers is comparable, with *T*_5%_ values above 343–344 °C. As observed, radiotherapy does not affect the *T*_5%_ values. The oxidative decomposition behavior of both implant layers is more complex than under inert conditions. The decomposition occurs in at least three to four stages, identified as *T*_max1_, *T*_max2_, *T*_max3_, and *T*_max4_ (Figure 9). These differences are directly related to the influence of oxygen on the activation energy of bond cleavage within the material structure. The presence of oxygen generally lowers the activation energy of degradation reactions, which is reflected in a shift in *T*_max_ values to lower temperatures compared to those observed under inert atmospheres [39,40,41,42,43,44,45,46,47,48,49,50]. At the same time, oxygen may initially promote the formation of more stable oxygen-containing intermediate species (oxygen–polymer interactions), which can temporarily increase thermal resistance at early degradation stages. However, with increasing temperature, oxidative processes dominate, leading to bond scission at lower *T*_max_ values. According to the literature, silicone materials heated above approximately 300 °C in the presence of oxygen undergo thermo-oxidative degradation, resulting in loss of physical properties and chemical breakdown. The organic side groups of the silicone chains are oxidized, the siloxane backbone is cleaved, and volatile and solid residues are formed [51,52]. At temperatures above approximately 450 °C, silicone materials may undergo auto-ignition, ultimately forming thermally stable residues such as silica (SiO_2_) or silicon monoxide (SiO). The present results confirm that radiotherapy does not significantly affect the thermo-oxidative decomposition behavior of the investigated silicone layers. Both shell and gel samples, before and after radiotherapy, exhibit similar thermal stability and decomposition stages occurring at comparable *T*_max_ values.

In contrast, the thermal stability of the prepared composites is significantly lower than that of the neat silicone implant wastes under oxidative conditions, by approximately 110 °C (Table 3, Figure 10). This decrease is directly associated with the presence of the PUM matrix, which exhibits moderate thermal stability (approximately 190 °C). The incorporation of PUM accelerates the thermo-oxidative degradation of the composites. On the other hand, the composites exhibit higher thermal stability than neat PUM by approximately 45 °C, indicating that the presence of silicone waste enhances the thermal resistance of the polymer matrix. PDMS acts as a thermal stabilizer, delaying the decomposition of the PUM matrix by forming a protective silica layer on its surface, which hinders the diffusion of oxygen into the polymer matrix. The simultaneous presence of PUM and silicone phases results in a more complex thermo-oxidative degradation process, occurring in at least five poorly resolved stages (*T*_max_). This suggests comparable energies of bond cleavage within the composite structure. Moreover, unlike PUM, which fully decomposes under oxidative conditions at around 600 °C, the composites do not completely decompose upon heating to 1000 °C. This is attributed to the formation of thermally stable residues resulting from the oxidative degradation of the silicone phases. However, the residual mass (*m*_r_) of the composites is lower than that of the neat shell or gel silicone materials. This indicates that PUM promotes further oxidation of silicone-derived residues at elevated temperatures. This effect is more pronounced for the shell–PUM composites, where the residual mass at 1000 °C is reduced by approximately 50%.

### 3.5. Gaseous FTIR in Oxidative Atmosphere

**Figure 11:**
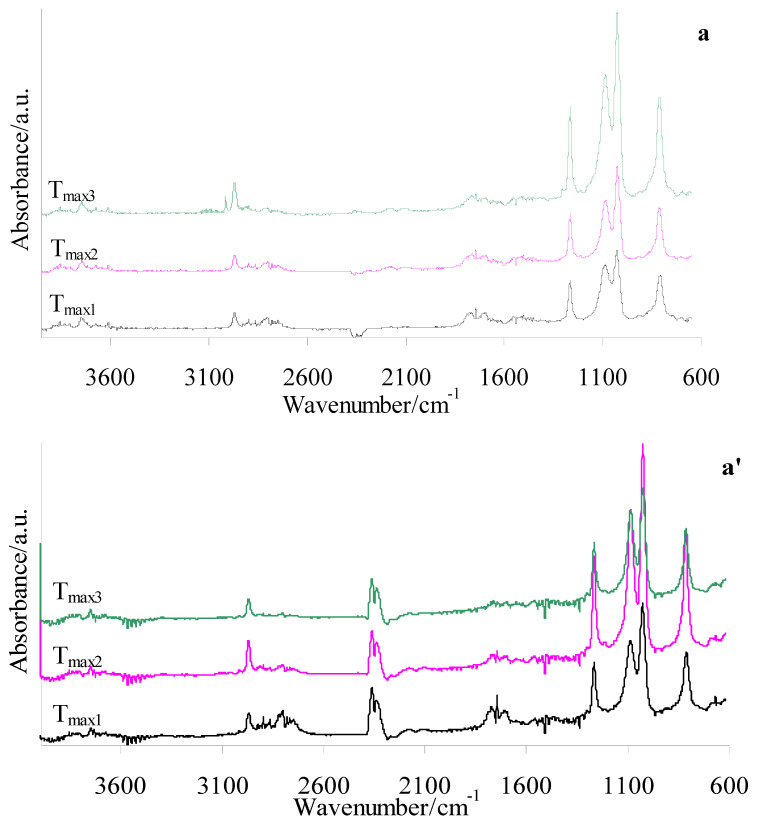

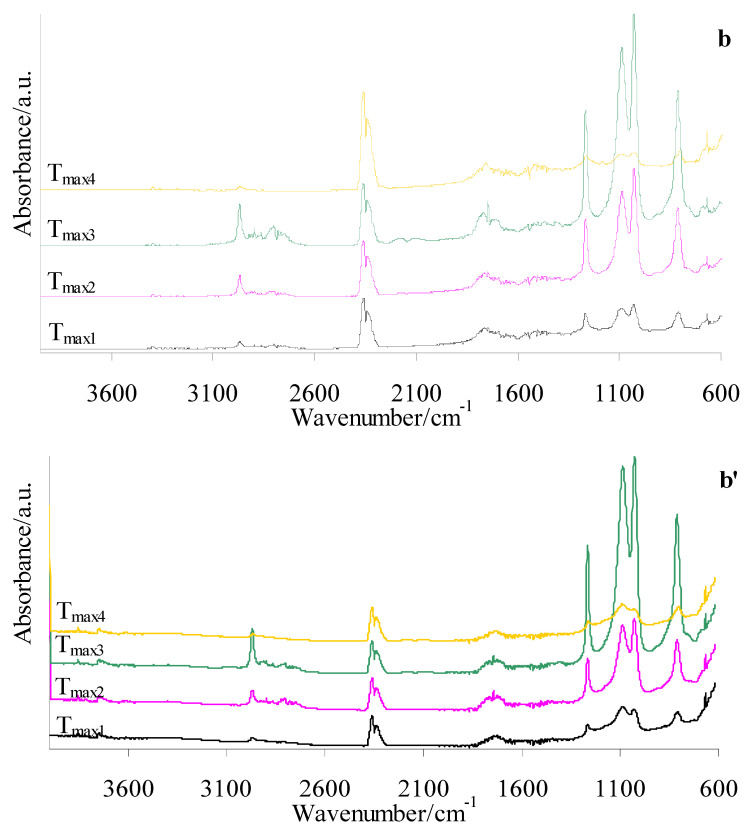
FTIR spectra of gaseous products released during heating of silicone implants in an oxidative atmosphere: shell before (a) and after radiotherapy (a’); gel before (b) and after radiotherapy (b’).

**Figure 12:**
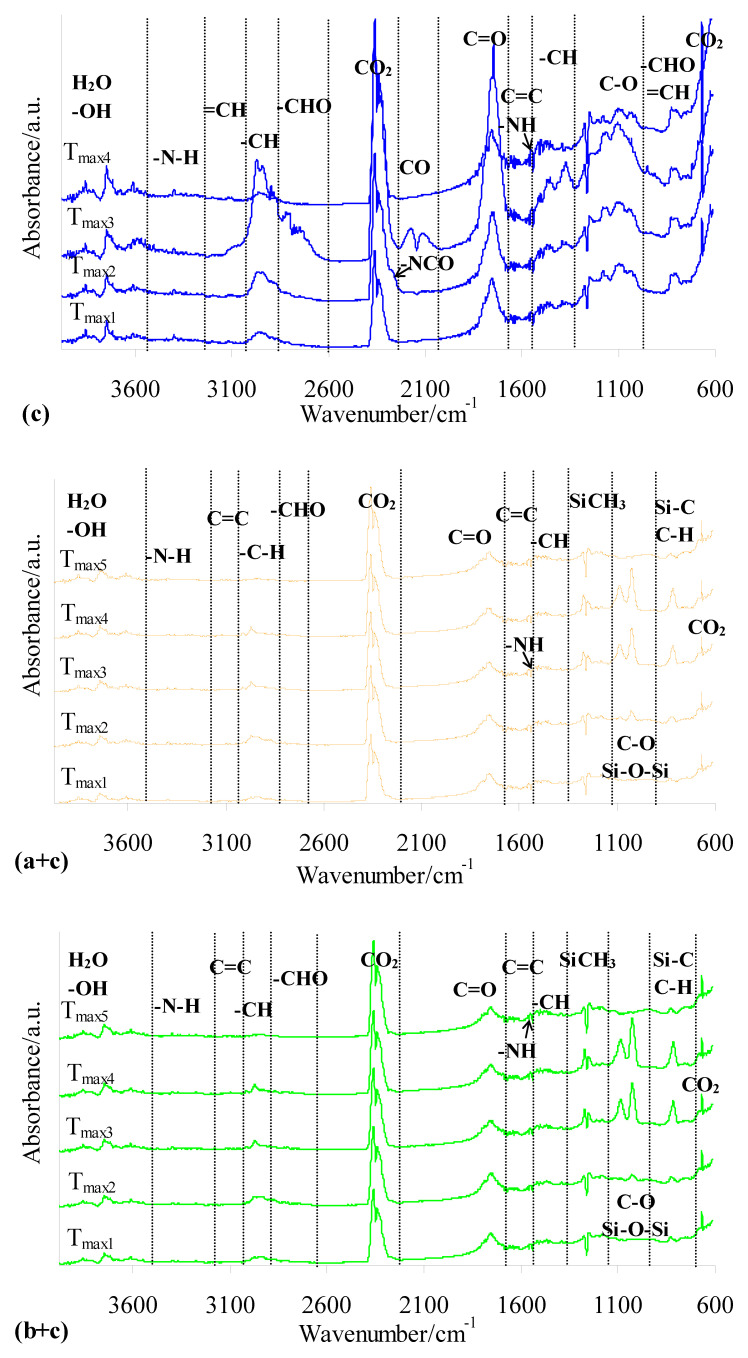
FTIR spectra of gaseous products released during heating of PUM (c) and the prepared composites: shell–PUM (a + c) and gel–PUM (b + c).

The FTIR spectra of gaseous products released during the oxidative decomposition of shell and gel silicone implants (before and after radiotherapy) (Figure 11) and the prepared composites and PUM (Figure 12) were collected to identify the emitted volatile compounds and assess their potential toxicity. The evaluation of gaseous products formed during heating in the presence of oxygen, particularly for the novel shell–PUM and gel–PUM composites, is important for assessing their behavior during combustion-based disposal after service life. As clearly observed, radiotherapy does not influence the type of volatile products emitted during the thermo-oxidative decomposition of either silicone implant layer. Both shell and gel fractions generate similar gaseous products. The FTIR spectra indicate that degradation involves oxidation of side groups, cross-linking and rearrangement reactions, and cleavage of siloxane bonds, leading mainly to the emission of low-molecular-weight cyclic siloxanes, silica-related species, CO, CO_2_, and water vapor (Figure 11), which is consistent with literature data [43,44,45,46,47,48,49,50,51,52,53,54,55].

In contrast, the FTIR spectra of PUM during oxidative degradation show similar absorption bands over the entire decomposition range, with maximum emission intensity observed at *T*_max3_. This indicates a simultaneous breakdown of bonds within the PUM structure, likely associated with its cross-linked network. Based on Figure 12 (c), the following characteristic vibrations are identified during oxidative decomposition of PUM: stretching vibrations of –OH and H_2_O (above 3600 cm^−1^), N–H stretching (3320–3390 cm^−1^), =CH stretching (3072–3091 cm^−1^), C–H stretching (2863–2964 cm^−1^), C–H stretching in aldehyde groups (2724–2760 cm^−1^), CO_2_ emission bands (2359–2310 cm^−1^ and 669 cm^−1^), isocyanate bands (2256 cm^−1^), CO emission bands (2092 cm^−1^ and 2163 cm^−1^), C=O stretching (1745 cm^−1^), C=C stretching (1627–1630 cm^−1^), N–H bending (1529 cm^−1^), C–H deformation (1388–1440 cm^−1^), C–O stretching (1020–1253 cm^−1^), and out-of-plane deformation vibrations of =CH and –CH groups in –CHO fragments (727–946 cm^−1^). The presence of these bands confirms the formation of urethane, aldehyde, alcohol, amide, organic acid, and isocyanate fragments, as well as inorganic gases (CO_2_, CO, and H_2_O) during oxidative decomposition of the PUM matrix used in the composites.

The FTIR profiles of both composite types (shell–PUM and gel–PUM) (Figure 12 (a + c and b + c)) are highly similar at corresponding *T*_max_ values, indicating the release of volatile products with comparable composition. At *T*_max1_ (200–220 °C), the emitted gases are primarily associated with the decomposition of the PUM matrix. In this region, only characteristic bands related to PUM degradation are observed, while no signals attributable to silicone-derived volatiles are detected. This suggests that, at this stage, mainly low-molecular-weight species and side groups of the PUM undergo oxidative decomposition. At higher temperatures (*T*_max2_), volatile products originating from both PUM and silicone implant waste are detected. The maximum emission intensity occurs at *T*_max3_ (380–394 °C) and *T*_max4_ (456–460 °C). At these stages, the spectra show bands corresponding to water vapor (above 3600 cm^−1^), low-intensity urethane-derived species (N–H at 3320–3390 cm^−1^ and 1529 cm^−1^), aldehyde fragments (C=O at 1745 cm^−1^ and C–H in aldehydes at 2724–2760 cm^−1^), and acid-related broad bands (CO and O–H around 3720 cm^−1^). Weak signals of unsaturated species (=CH at 3072–3091 cm^−1^ and C=C at 1627–1630 cm^−1^) are also observed, along with emissions of cyclic siloxanes and silica-related species (Si–CH_3_ at 1267 cm^−1^, Si–O–Si at 1022–1076 cm^−1^, and Si–CH_3_ at 815 cm^−1^), as well as CO_2_ [19]. Notably, no significant formation of CO or isocyanates is detected in this temperature range (Figure 12).

In summary, the oxidative decomposition of the prepared composites is a complex process involving multiple simultaneous reactions, including bond cleavage, secondary reactions between intermediate products and oxygen, and the formation of CO_2_ and H_2_O. In addition, high-temperature sintering processes of the residual solid phase occur, leading to the formation of thermally stable residues. Compared to the residues formed from neat silicone implants, the residual mass of the composites is reduced, indicating enhanced degradation efficiency in the composite systems.

### 3.6. Dynamic Mechanical Analysis (DMA) and Brinell Hardness

**Figure 13:**
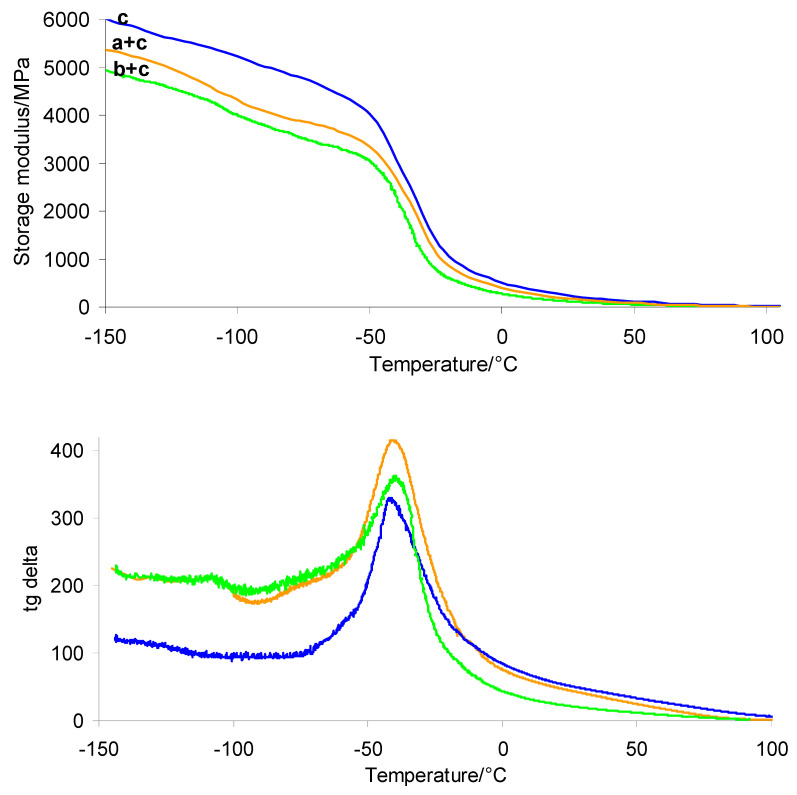
Storage modulus and tg delta of PUM (c) and the prepared composites: shell–PUM (a + c) and gel–PUM (b + c).

The results obtained from DMA and Brinell hardness measurements are presented in Table 4. Additionally, the storage modulus and tan *δ* curves as a function of temperature are shown in Figure 13. The DMA study indicates that both the PUM matrix and the prepared shell–PUM and gel–PUM composites are flexible materials. However, the incorporation of silicone breast implant waste into the polymer composites (shell–PUM and gel–PUM) further reduces their stiffness. The incorporation of silicone waste into the composites leads to a decrease in the storage modulus at room temperature (*E*_20°C_) compared to that of the pure PUM matrix. The PUM matrix exhibits an *E*_20°C_ value of 280.2 MPa, whereas the composites show values of 206.7 MPa (shell–PUM) and 139.2 MPa (gel–PUM). This suggests that silicone breast implant waste acts as an internal lubricant, particularly the gel layer, enhancing polymer chain mobility and thereby increasing the flexibility of the resulting composites.

DMA also confirms phase separation, as two distinct relaxation processes are observed in the tan *δ* curves, corresponding to the glass transitions of the silicone phase (at lower temperatures) and the polymer matrix (at higher temperatures). However, the tan *δ* peak maximum of the PUM phase in the composites is slightly shifted toward higher temperatures (ca. −39 °C) compared to that of the pure PUM matrix (−44.2 °C). This indicates the presence of interactions between the polymer matrix and the silicone phase, i.e., modification of polymer chain mobility at the phase boundary.

The Brinell hardness of pure PUM is 23.3 ± 1.2 MPa. In contrast, the composites exhibit significantly lower hardness values: 10.8 ± 0.5 MPa for the shell–PUM composite and 9.8 ± 0.3 MPa for the gel–PUM composite. Brinell hardness analysis reveals a substantial decrease in indentation resistance following the incorporation of dispersed shell and gel silicone phases into the PUM matrix. This reduction of over 50% confirms the plasticizing effect of the silicone waste on the polymer network. Silicone acts as a flexible filler, reducing the overall stiffness of the polymer matrix, which in turn lowers both the surface hardness and the glass transition temperature of the shell–PUM and gel–PUM composites. Flexible silicone chains interact with the matrix by penetrating between polymer chains, leading to a loosening of the PUM structure. Consequently, the presence of shell or gel phases enhances the elasticity of the composite material.

### 3.7. Microscopic and Profilometric Analysis

**Figure 14:**
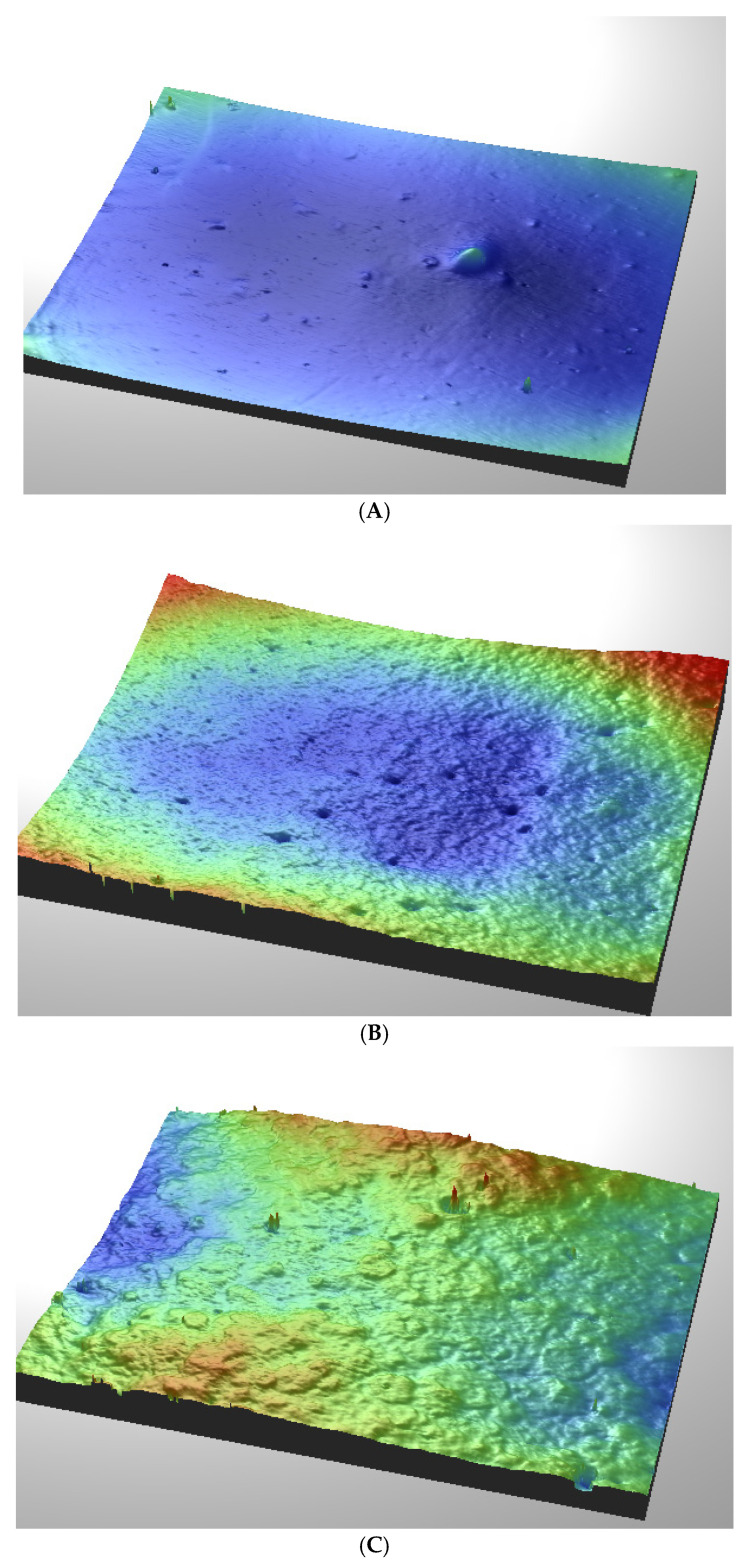
Three-dimensional surface topography of the sample obtained using a Contour GT-K1 optical profilometer (Veeco). (**A**) PUM (c); the prepared composites: (**B**) shell–PUM (a + c) and (**C**) gel–PUM (b + c).

Figure 14 shows that the three-dimensional surface topography analysis reveals noticeable differences in the morphology and roughness of the tested samples. The unmodified PUM sample exhibited the lowest average surface roughness (Ra = 177 nm), whereas the shell–PUM and gel–PUM composites exhibited increased roughness values of 271 and 309 nm, respectively. These results suggest that incorporating shell and gel modifiers results in a more heterogeneous and rougher surface structure.

## 4. Conclusions

In this work, the effect of radiotherapy on the structure and properties of silicone implants composed of two layers (shell and gel) was investigated. Moreover, the reuse of silicone implants as a component of layered composites was proposed. The conducted analyses confirmed that radiotherapy does not affect the structure, glass transition temperature, melting and crystallization behavior, degree of crystallinity, thermal stability, or thermo-oxidative degradation pathway of the investigated silicone implants. The shell and gel layers of silicone implant waste were successfully incorporated as components of layered composites with PUM. The resulting composite materials exhibited two glass transition regions, both below room temperature: one at approximately −117 to −119 °C and the other at approximately −61 °C. The composites showed a more amorphous structure compared to the silicone implant waste. The ability to crystallize decreased significantly, from approximately 49–50% (shell) to 22.1% (shell–PUM composite) and from approximately 87.1–87.5% (gel) to 33.4% (gel–PUM composite). This effect is attributed to steric hindrance imposed by the PUM matrix, interfacial interactions, and the reduced proportion of silicone waste in the overall composition. DMA confirmed the increased elasticity of the obtained composites compared to the pure PUM matrix. The determined Brinell hardness of the silicone waste-based composites was lower than that of neat PUM, which is attributed to the plasticizing effect of the shell and gel phases within the composites. The introduction of shell and gel additives increases the surface roughness of PUM composites, suggesting a less uniform surface structure than the unmodified material. The thermal stability of the composites was approximately 40 °C higher than that of the PUM matrix. The thermal stability of the shell–PUM and gel–PUM composites was approximately 222.5–226.8 °C under inert conditions and 235–237 °C under oxidative conditions. The thermo-oxidative degradation behavior of the prepared silicone waste-based composites was more complex compared to that of both silicone implant waste and PUM. As confirmed, oxidative degradation involves multiple simultaneous processes leading to the emission of a mixture of inorganic and organic volatiles, including urethane, aldehyde, alcohol, amide, organic acid, unsaturated fragments, CO_2_, and H_2_O. These results not only demonstrate the effect of radiotherapy on the structure and properties of silicone implants but, more importantly, present a potential approach for the valorization of silicone implant waste and the development of composite materials with tailored properties for practical applications.

## Figures and Tables

**Table 1:** DSC data.

Sample	*T*_g_/°C	*T*_m_/°C	Δ*H*_m_/J/g	*T*_c_/°C	Δ*H*_f_/J/g	*χ*/%
a	−123.9	−36.6	30.92	−71.6	30.55	49.9
a’	−120.8	−39.5	30.53	−72.1	30.30	49.5
b	−121.3	−42.3	55.56	−78.9	53.54	87.5
b’	−120.3	−39.5	55.33	−78.2	53.29	87.1
a + c	−119.4/−60.9	−37.7	13.70	−72.2	13.55	22.1
b + c	−117.6/−61.1	−39.5	20.90	−84.7	20.10	33.4
c	−50.8	-	-	-	-	-

**Table 2:** TG/DTG data in inert atmosphere.

Sample	*T*_5%_/°C	*T*_max1_/*T*_max2_/*T*_max3_/*T*_max4_/*T*_max5_/°C	Δ*m*_1_/%	*m*_r_/%
a	330.8	529.2/639.5/717.2	63.2	36.8
a’	330.9	530.5/639.8/721.1	63.8	36.2
b	330.1	411.1/487.7	100	0
b’	330.5	415.4/477.9	100	0
a + c	226.8	221.5/360.9/405.6/514.8/665.3	92.9	7.1
b + c	222.5	220.6/345.5/419.4/500.3/661.8	100	0
c	195.3	222.4/381.6/541.5	100	0

*m*_r_—residual mass at 1000 °C.

**Table 3:** TG/DTG data in oxidative atmosphere.

Sample	*T*_5%_/°C	*T*_max1_/*T*_max2_/*T*_max3_/*T*_max4_/*T*_max5_/°C	Δ*m*_1_/%	*m*_r_/%
a	344.2	356.8/429.8/499.7	56.1	43.9
a’	344.5	368.4/428.3/500.1	59.9	40.1
b	343.2	351.2/383.2/430.8/507.3	72.8	27.2
b’	343.0	352.8/383.7/428.5/519.2	82.9	18.1
a + c	237.2	200.2/327.3/394.1/456.5/539.2	77.5	22.5
b + c	235.8	220.4/340.1/380.2/460.3/540.5	89.8	10.2
c	190.0	215.1/310.4/386.6/565.7	100.0	0

*m*_r_—residual mass at 1000 °C.

**Table 4:** DMA data and Brinell hardness for PUM, shell–PUM and gel–PUM composites.

Sample	*E’*_20°C_/MPa	tg*δ*/°C	HK/MPa
a + c	206.7	−107.5/−39.2	10.8 ± 0.5
b + c	139.2	−107.3/−39.0	9.8 ± 0.3
c	280.2	−44.2	23.3 ± 1.2

## Data Availability

The original contributions presented in this study are included in the article. Further inquiries can be directed to the corresponding author.
